# Preventive Strategies for Driveline Infections in Ventricular Assist Devices: A Systematic Review

**DOI:** 10.1097/MAT.0000000000002800

**Published:** 2026-08-03

**Authors:** Simone Amato, Francesco Limonti, Andrea Battisti, Antonio Cartolano, Angela Lappa, Emilio D’Avino, Guglielmo Imbriaco

**Affiliations:** From the * Cardiac Intensive Care Unit, Heart Transplant Centre and ECMO Service, San Camillo Forlanini Hospital, Rome, Italy; † Department of Pharmacy, Health and Nutritional Sciences (DFSSN), University of Calabria, Rende, Italy; ‡ Cardiovascular Perfusionist, Azienda Ospedaliero-Universitaria Sant’Andrea di Roma, Rome, Italy; § Emergency and Critical Care Department, Policlinico Umberto I, Rome, Italy; ¶ Department of Heart and Vessels, Cardiovascular Intensive Care Unit, San Camillo Forlanini Hospital, Rome, Italy; ‖ Department of Health Professions, San Camillo Forlanini Hospital, Rome, Italy; # Emergency Medical Communication and Prehospital Emergency Service (118 Emilia Est), Maggiore Hospital Carlo Alberto Pizzardi, Bologna, Italy.

**Keywords:** driveline infection, infection prevention, mechanical circulatory support, surgical wound infection, ventricular assist device

## Abstract

Driveline infection (DLI) is the most frequent infectious complication in patients supported with ventricular assist devices (VADs) and remains a major cause of morbidity and rehospitalization. This systematic review aimed to synthesize the available evidence on preventive strategies to reduce the incidence of DLI in adult VAD recipients. A systematic search of MEDLINE/PubMed, Scopus, CINAHL, Web of Science, the Cochrane Library, and APA PsycInfo up to April 1, 2026, followed Preferred Reporting Items for Systematic Reviews and Meta-Analyses (PRISMA) 2020 guidelines. Eleven observational studies were included. The infection prevention strategies evaluated included modifications to surgical tunneling, driveline stabilization techniques, standardized exit-site care protocols, antiseptic and dressing management, negative pressure wound therapy, and antibiotic prophylaxis. Surgical routing optimization, mechanical stabilization, and standardized exit-site care protocols were most consistently associated with reduced DLI incidence. In contrast, adjunctive interventions such as negative pressure wound therapy and prolonged antibiotic prophylaxis showed limited or inconsistent benefit. Current evidence remains limited to observational studies, highlighting the need for prospective and controlled research to establish standardized preventive strategies.

The use of left ventricular assist devices (LVADs) has become an established therapeutic option for patients with advanced heart failure in a highly selected patient population, serving both as a bridge to transplantation and as destination therapy. Despite improvements in device design and patient management, device-related complications remain a significant clinical challenge, with infections representing one of the most frequent and impactful adverse events in this population.^1,2^

Among LVAD-related infections, driveline infection (DLI) is the most common clinical manifestation. Driveline infections have been reported to occur in approximately 34.7% of patients supported with continuous-flow LVADs.^3^ The percutaneous driveline creates a permanent transcutaneous tract, disrupting the skin barrier and providing a portal of entry for microorganisms. This mechanism predisposes patients to local infections that can extend along the tunnel tract and, in some cases, lead to systemic complications. Driveline infections contribute substantially to morbidity, mortality, prolonged hospitalization, and increased healthcare utilization, as reported in observational studies^3,4^ in patients supported by mechanical circulatory devices.^5,6^ More complex DLIs, particularly those involving the pump or pocket, or associated with systemic involvement, may significantly worsen prognosis, and in selected refractory cases, heart transplantation may represent a salvage option.^4^

The pathogenesis of DLI is multifactorial.^7^ Risk factors include patient comorbidities, duration of support, device characteristics, and exit-site management practices.^8^

To mitigate this burden, a range of preventive strategies have been implemented across centers, including modified surgical tunneling techniques, standardized exit-site care packages, antiseptic regimens, driveline stabilization systems, and structured patient education programs.^9,10^ However, preventive approaches vary considerably between institutions, and reported outcomes remain heterogeneous. No universally accepted standard for DLI prevention has been established.^9,11^ In addition, variability in infection reporting and clinical presentation limits comparability across studies.^12^ No randomized controlled trials specifically evaluating preventive interventions for DLI were identified, and the available evidence derives exclusively from observational cohorts and quality improvement initiatives. Consequently, uncertainty persists regarding which preventive strategies are most effective in reducing the incidence of DLI and which components of care have the greatest clinical impact.

The objective of this systematic review was to synthesize and critically appraise the available evidence on preventive strategies aimed at reducing the incidence of DLI in adult patients supported with ventricular assist devices.

## Methods

This systematic review was conducted following the Preferred Reporting Items for Systematic Reviews and Meta-Analyses (PRISMA) 2020 guidelines.^13^ The review protocol was registered in the International Prospective Register of Systematic Reviews (PROSPERO; registration number CRD420251184902).

The specific search strategy was developed around the following research question, based on the PICO framework (Population, Intervention, Comparison, Outcomes): “In adult patients with ventricular assist devices, which preventive strategies may reduce driveline infections?”

### Population

Studies were considered eligible if they included adult patients (≥18 years) supported with durable ventricular assist devices, including LVAD, right ventricular assist devices (RVAD), or biventricular assist devices (BiVAD). Studies on pediatric populations or patients supported with temporary mechanical circulatory support systems were excluded.

### Interventions

The review included studies evaluating preventive strategies for DLI. Eligible interventions encompass antisepsis and skin preparation protocols (*e.g*., chlorhexidine-based regimens or decolonization strategies), driveline exit-site dressings and dressing-change frequency, securement or anchoring devices, modified fixation or tunneling techniques (including double or triple tunneling), silicone–skin interfaces, intraoperative protective barriers (*e.g*., extracellular matrix scaffolds), standardized care bundles, structured education programs for patients or caregivers, and peri- or postoperative antibiotic prophylaxis aimed at primary prevention. Studies focusing on the treatment of established DLI were excluded.

### Comparators

Eligible studies included a comparator, defined as usual care or an alternative preventive strategy. Both parallel group comparisons and before-after designs with historical or concurrent controls were considered.

### Outcomes

The primary outcome of interest was the incidence of DLI, reported as proportions, incidence rates, or time-to-event measures, using definitions provided by study authors (with preference for VAD-specific or International Society for Heart & Lung Transplantation [ISHLT] definitions, when available).

Secondary outcomes included deep or pocket infection, bloodstream infection secondary to DLI, device exchange or explantation due to infection, and infection-related hospitalizations, as well as other device-related infectious complications when explicitly related to DLI by study authors.

The definitions of DLI and infection-related outcomes used in the included studies are summarized in Table 1.

**Table 1.** Definitions of Driveline Infection and Infection-Related Outcomes Across the Included Studies

Study	Definition of DLI/Outcome	Diagnostic Criteria	Classification System Used	Notes
Balestra *et al*. (2023)^14^	Local infection at the driveline exit site within 30 d after surgery	Identified by clinical signs and confirmed by leukocytosis or elevated C-reactive protein	Author-defined/study-specific	Source material available for this table did not provide a verbatim formal case definition
Cagliostro *et al*. (2016)^15^	Percutaneous site and—or pocket infection	A positive culture from the skin and/or tissue surrounding the driveline or pump housing, coupled with the need for antimicrobial therapy, in the presence of clinical evidence of infection such as pain, fever, drainage, or leukocytosis	INTERMACS	Explicit definition stated in Methods
Camboni *et al*. (2017)^16^	Driveline exit-site infection	A positive culture from the skin and/or tissue surrounding the driveline, coupled with the need for antimicrobial therapy when there was clinical evidence of infection (pain, fever, drainage, or leukocytosis)	INTERMACS	Explicit definition stated in Methods
Durand *et al*. (2017)^17^	DLI and DLI-related bacteremia/pump or pocket infection	DLIs were identified by record review and adjudicated according to published criteria (ISHLT). Superficial DLI involved tissues superficial to fascial and muscle layers; deep DLI involved fascial and muscle layers. Cases with drainage alone that resolved with local measures were not considered DLIs. Superficial DLIs with skin flora only were included as culture-negative. Pump/pocket infection due to retrograde extension or DLI-related bacteremia was considered DLI-related	ISHLT-based adjudication	Most detailed operational definition among the included studies
Fleissner *et al*. (2013)^18^	DLI within 1 y after implantation	The accessible full text reported 1 y driveline infection rates but did not provide a detailed operational diagnostic definition in the reviewed text used to prepare this table	Not explicitly stated in accessible text	Outcome appears author-defined
Lauenroth *et al*. (2023)^19^	Driveline infection	The article specified DLI as the primary endpoint, but the accessible text reviewed for this table did not include a detailed operational diagnostic definition	Not explicitly stated in accessible text	Study conducted within a standardized in-house driveline care protocol
Matsumoto *et al*. (2018)^20^	Driveline infection and deep driveline infection	A positive bacterial culture obtained from the skin and/or tissue surrounding the driveline, coupled with the need for antimicrobial therapy in patients with clinical evidence of infection such as pain, fever, exudation, or leukocytosis, in accordance with the INTERMACS definition of percutaneous site infection. Diagnosis was made at initiation of antimicrobial therapy or when a positive culture was obtained. Patients without a positive culture or antimicrobial therapy were excluded even if the exit site was wet or painful. Deep infection was defined as extension >3 cm from the exit site diagnosed by CT or Ga-67 scintigraphy	INTERMACS	Explicit definition stated in Methods
Schibilsky *et al*. (2011)^21^	Late-onset driveline exit-site infection or superficial driveline exit-site infection	The accessible full text described rates of superficial exit-site infection and revision surgery, but did not provide a detailed operational diagnostic definition in the reviewed text used to prepare this table	Not explicitly stated in accessible text	Outcome appears author-defined
Seretny *et al*. (2022)^22^	First DLI within 1 y after implant	Defined according to INTERMACS definitions, including clinical symptoms accompanied by a positive culture and initiation of antibiotic treatment	INTERMACS	Explicitly reported in Methods
Tsuji *et al*. (2022)^23^	Driveline infection within 1 y after LVAD implantation	The article reported DLI as the primary outcome within 1 y after implantation. The accessible text reviewed for this table did not provide a more detailed operational diagnostic definition	Not explicitly stated in accessible text	Outcome assessed comparatively in NPWT *vs*. standard dressing groups
Yoshitake *et al*. (2018)^24^	Driveline infection requiring treatment or readmission	Diagnosed according to the International Society of Heart and Lung Transplantation consensus: clinical evidence of infection (redness, swelling, pain, fever, fluid discharge, or leukocytosis) associated with a positive culture from the skin and/or tissue surrounding the driveline. Additionally, the study defined DLI as cases requiring readmission for intravenous antimicrobial therapy or debridement	ISHLT consensus	Explicit definition stated in Methods

Definitions are transcribed or conservatively summarized from the accessible full texts and extracted study materials reviewed for this project. Where the reviewed text did not state a verbatim operational definition, this is explicitly indicated.

CT, computerized tomography; DLI, driveline infection; INTERMACS, Interagency Registry for Mechanically Assisted Circulatory Support; ISHLT, International Society for Heart and Lung Transplantation; LVAD, left ventricular assist devices; NPWT, negative pressure wound therapy.

### Study Designs and Settings

Randomized controlled trials, quasi-experimental studies, prospective or retrospective cohort studies, before-and-after studies, interrupted time-series analyses, and quality improvement studies with comparative data were included. Case reports, single-arm case series without comparators, purely epidemiological or risk factor studies, *in vitro* or biomechanical studies, guidelines, consensus statements, and qualitative studies without clinical outcomes were excluded.

Studies conducted in perioperative, outpatient, or home care settings were considered eligible.

### Information Sources and Search Strategy

A comprehensive search was conducted in MEDLINE/PubMed, Scopus, Cumulative Index to Nursing and Allied Health Literature (CINAHL), Web of Science, the Cochrane Library, and American Psychological Association (APA) PsycInfo for records indexed up to April 1, 2026. Gray literature was searched using Google Scholar.

Relevant records were screened, and additional sources were considered when full texts were available. No date or language restrictions were applied. The full electronic search strategies for each database are reported in Supplementary File 1, Supplemental Digital Content 1, https://links.lww.com/ASAIO/C59.

### Selection Process

The extracted records were uploaded to Rayyan.^25^ After the removal of duplicates, an initial screening assessment was conducted independently at the title and abstract level by two investigators (F.L. and A.C.).

The final selection of studies was based on the evaluation of the complete manuscripts by the same two investigators, independently assessing the full texts of potentially eligible articles to determine final inclusion. The reference lists of both included and excluded full-text articles were manually screened for additional potentially relevant studies not identified through the electronic database searches. Reasons for exclusion at the full-text stage were discussed and documented. At any stage of the selection process, disagreements were resolved by discussion.

### Data Extraction and Quality Assessment

Data extraction was independently performed by two reviewers (A.L. and E.D.) using a standardized extraction form; discrepancies were resolved through discussion. Extracted data included: author(s) and year of publication, country, study aims or purpose, population characteristics, study methodology, reported outcomes, key findings, and level of evidence.

Quality assessment and risk of bias appraisal were conducted independently by two investigators (S.A. and A.B.). The methodological quality of the included studies was evaluated using the Joanna Briggs Institute (JBI) Critical Appraisal Tools,^26^ selecting the version appropriate to each study design. The level of evidence of the included studies was also classified according to the Oxford Center for Evidence-Based Medicine (OCEBM) framework. The certainty of evidence for each intervention category was assessed using the Grading of Recommendations Assessment, Development, and Evaluation (GRADE) approach.

Disagreements in quality assessment were resolved through discussion and, if necessary, involving a third investigator. The results of the risk of bias assessments were not used as exclusion criteria but were considered in the interpretation of findings, sensitivity analyses, and grading of the certainty of evidence.

## Results

The database search identified 1,046 records across all sources. After removal of 262 duplicates, 784 records remained for title and abstract screening. Of these, 658 records were excluded, and 120 full-text articles were assessed for eligibility. Following full-text evaluation, 109 studies were excluded for not meeting the inclusion criteria. Ultimately, 11 studies were included in the qualitative synthesis. The study selection process is shown in Figure 1.

**Figure 1.**
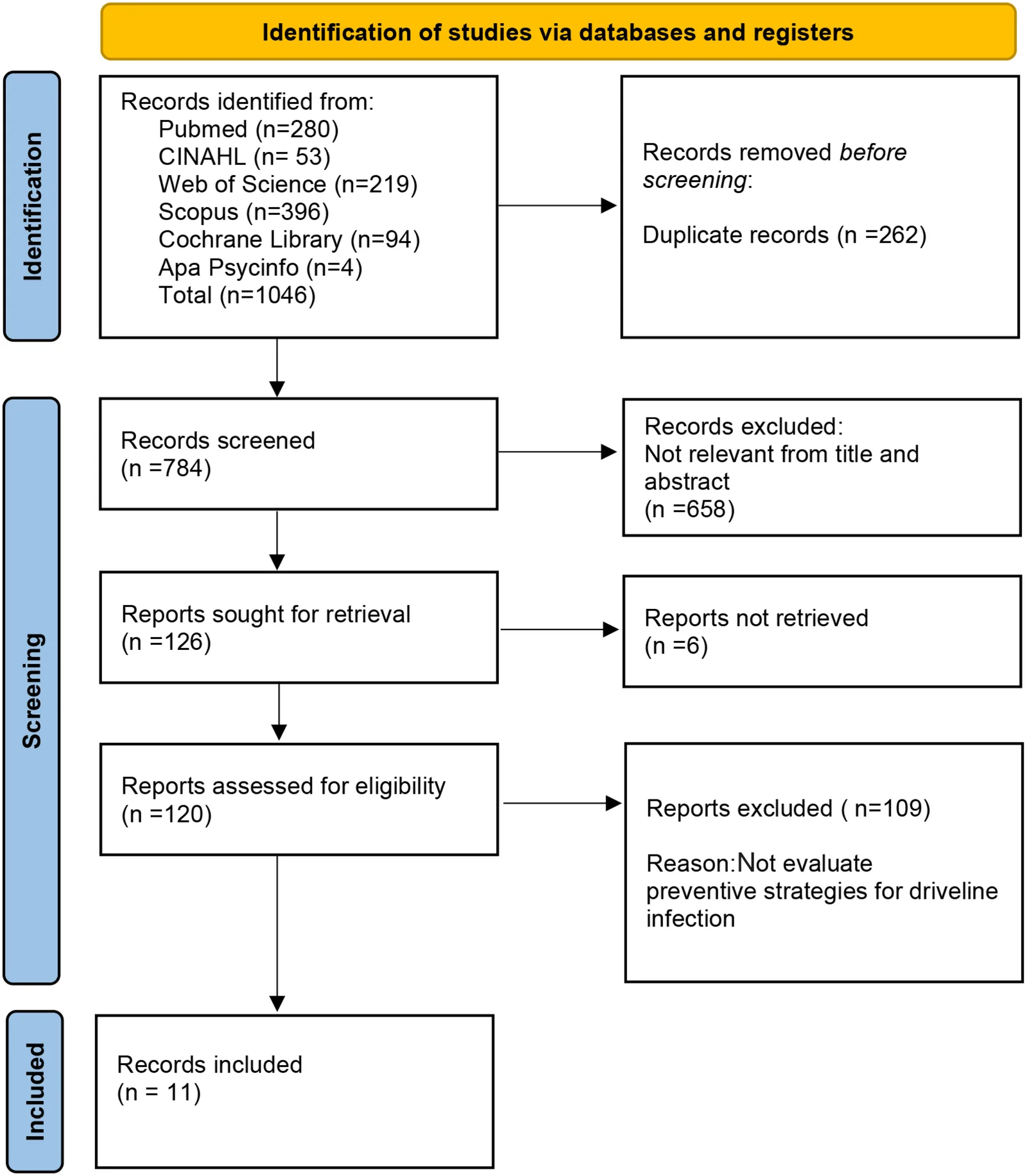
PRISMA flowchart of the record selection process. PRISMA, Preferred Reporting Items for Systematic Reviews and Meta-Analyses (https://www.prisma-statement.org/prisma-2020-flow-diagram).

### Study Characteristics

A total of 11 studies met the inclusion criteria and were included in the qualitative synthesis.^14–24^ The studies, published between 2011 and 2023, were conducted across five countries: Germany,^16,18,19,21^ Japan,^20,23,24^ the United States,^15,17^ Brazil,^14^ and Canada.^22^

All included studies employed observational designs. The majority (five) were retrospective cohort studies,^14,17,18,21,24^ while others were prospective cohort or observational studies.^15,16,19,20^ One study used a quasi-experimental comparative design to evaluate a perioperative preventive strategy,^23^ and one was reported as a cross-sectional quality improvement study with comparative elements.^22^

All studies focused on adult patients supported with LVADs and evaluated preventive strategies targeting DLI. The interventions assessed included surgical tunneling modifications,^18,24^ standardized care bundles and structured exit-site protocols,^14,21^ antiseptic and dressing management approaches,^14,19–21^ prophylactic negative pressure wound therapy (NPWT),^23^ and topical or systemic antibiotic strategies.^17^ Silicone-based exit-site strategies^16^ were included within antiseptic and dressing-related approaches. The preventive interventions reported in the included studies can be broadly categorized into three main domains: surgical or device-related strategies, driveline stabilization and securement techniques, and exit-site care protocols, including antiseptic regimens and dressing management approaches.^14,19–22^ An overview of the included studies, including preventive interventions, key findings, LVAD devices evaluated, and reported patient-related risk factors, is provided in Table 2.

**Table 2.** Summary of Included Studies

Author(s), Year (Country)	Aims/Purpose	Population	Study Design	Outcomes	Key Findings	Levels of Evidence	Device(s) Included	Patient-Related Risk Factors Reported
Balestra *et al*. (2023) (Brazil)^14^	To evaluate the different types of dressings used for the driveline exit site in HM patients and to describe the incidence density of local complications (infections, bleeding, and pressure ulcers) in the 30 d following surgery	22 patients; median age 53.5 y	Retrospective cohort study	Infection (identified by clinical signs and confirmed by leukocytosis or elevated CRP). Bleeding and pressure ulcers associated with the medical device. Variability is observed in the adopted dressing protocols	Extreme variability, with 22 different dressing types identified. Type 6 dressing (Chlorhexidine, Excilon, gauze, and IV3000 transparent film) was the most commonly used (45.4%). Infection was the most common complication (45.4%). Bleeding was the second most common complication (27.7%) and was significantly associated with increased duration of extracorporeal circulation (CPB) during the procedure. Use of the Hollister anchoring device was associated with higher rates of infection (61.1%) and pressure ulcers (11.1%) compared with other methods	2	HeartMate II; HeartMate III	Age, sex, INTERMACS, CPB duration, surgery duration, device type. No significant association with infection; CPB duration associated only with bleeding
Cagliostro *et al*. (2016) (United States)^15^	To determine whether the implementation of a standardized drive line care kit can reduce DLI in LVAD patients	266 patients; mean age 58.7 ± 13.2 y. Group A (n = 107); Group B (n = 159)	Prospective cohort study	Outcomes included DLI event rate per patient-year, 1-y infection freedom, hospital readmission rates, and mortality	The driveline infection rate decreased from 0.18 to 0.07 events per patient-year. Relative risk reduction of 62.5% and absolute risk reduction of 11%. One year freedom from DLI increased significantly from 81.94% to 92.46%. The readmission rate for DLI decreased from 0.08 to 0.03 events per patient-year	3	HeartMate II; HVAD	BSA, age, sex, smoking, HTN, DM, chronic renal insufficiency reported; subgroup analysis included gender, BSA, age, strategy and diabetes
Camboni *et al*. (2017) (Germany)^16^	To evaluate whether a silicone skin-level interface could reduce the risk of infection at the DL exit site compared with other materials (*e.g*., velour)	40 patients; mean age 54.4 y. Silicone group (n = 16; mean age 57.1 y); Velour group (n = 24; mean age 52.6 y)	Prospective, observational study	Infection rate: In the Velour group, 6 patients (25%) developed an infection *vs*. 1 patient (6%) in the silicone group. The event rate per patient-year (EPPY) was 0.10 for silicone versus 0.34 for velour (*p* = 0.30). One year survival was similar between the two groups (69% silicone *vs*. 75% velour, *p* = 0.67)	The use of silicone at the driveline exit site was associated with fewer infections. The silicone group showed a trend toward lower infection rates despite having patients with a higher preoperative risk profile, such as elevated BMI and CRP. The silicone material was easier to clean than velour, reducing the potential for pathogens to adhere to the surface	3	INCOR LVAD	BMI, BSA, diabetes, renal dysfunction, CRP; silicone group had higher BMI/BSA, renal dysfunction, and CRP
Durand *et al*. (2017) (United States)^17^	To evaluate the effect of topical polymyxin–trimethoprim prophylaxis on the incidence of DLI in patients with CF-LVADs	84 patients; mean age 55 y. Poly group (n = 65; mean age 55 y); no-poly group (n = 19; mean age 53 y)	Retrospective cohort study	Incidence of superficial and deep DLI and DLI-related bacteremia and infection-free survival	Prophylaxis reduced the overall incidence of DLI by 75% and the incidence of deep DLI by 95%	3	Continuous-flow LVADs; specific device types not listed in extracted table	Diabetes, BMI, obesity, overweight/obesity, BSA, albumin, destination therapy, support duration >1 y
Fleissner *et al*. (2013) (Germany)^18^	To test whether a surgical implantation technique based on double driveline tunneling can reduce the incidence of infections in patients with LVADs	81 patients; mean age 52.8 y. Group 1 (n = 40; mean age 52.8 y); Group 2 (n = 41; mean age 51.3 y)	Retrospective analysis comparing two groups	The 1 y infection rate was significantly reduced (22.5% in group 1 *vs*. 4.9% in group 2). There was no significant difference in overall 1 y mortality between the two groups. The total operating time was significantly longer in group 2 (210.78 *vs*. 166.1 min)	One year driveline infection rate was significantly reduced from 22.5% in group 1 to 4.9% in group 2 (*p* < 0.001). The double-tunneling implantation technique was identified as an independent predictor of reduced DLI risk. No significant difference in 1 y mortality was observed between groups (25.0% *vs*. 24.4%)	2	HVAD/HeartWare	LVEF, obesity/BMI >25, FFP use associated with DLI in univariate analysis; CKD, smoking, dialysis, COPD not significant
Lauenroth *et al*. (2023) (Germany)^19^	To investigate the effectiveness of preventive use of cold argon plasma (ACP) in reducing the risk of DLI in patients with LVAD	80 patients; median age 61 y. ACP group (n = 40; median age 63 y); control group (n = 40; median age 60 y)	Prospective study	ACP group no. 40 Control group no. 40 Primary endpoint: Incidence of DLI. Secondary endpoint: Overall survival during the follow-up period within 1 y	Preventive use of ACP significantly improved freedom from DLI at 30 d (100% in the ACP group *vs*. 85% in the control group) and at 6 mo (92% *vs*. 69%). At 1 y, freedom from DLI did not show statistically significant differences between the two groups (55% ACP group *vs*. 60% control group), suggesting the absence of a long-term prophylactic effect beyond the application period. Survival at 1 y tended to be higher in the ACP group (90%) than in the control group (74%), although the value did not reach statistical significance (*p* = 0.09)	3	HeartWare; HeartMate 3	Diabetes mellitus, hemofiltration, previous cardiac surgery, EUROMACS, NYHA class, eGFR, CRP reported as baseline variables
Matsumoto *et al*. (2018) (Japan)^20^	To explore the relationship between driveline exit direction and angle and the occurrence of driveline infections after LVAD implantation	71 patients; mean age 45 y	Prospective cohort study	10 patients with driveline infection (14%). Infection-free rates were achieved in 96% of patients at 6 mo, 88% at 12 mo, and 86% at 24 mo. The cutoff for the driveline angle was 41°	A driveline angle greater than 42° is an independent risk factor for infection (hazard ratio 4.71). Patients with an angle <41° showed significantly higher infection-free rates (90% at 24 mo) than those with an angle >42° (74% at 24 mo)	3	HeartMate II	Main evaluated factor: driveline angle; angle >42° was an independent risk factor. Patient-related predictors not central
Schibilsky *et al*. (2011) (Germany)^21^	To evaluate whether the double-tunnel technique can reduce the incidence and severity of exit site infections compared with the conventional technique	43 patients; mean age 55.7 y. Conventional group (n = 11; mean age 57.2 y); double-tunnel group (n = 32; mean age 55.2 y)	Retrospective cohort study	Number of superficial infections and revision surgeries; infection-free survival	The double-tunnel group showed a lower infection rate (0.638 *vs*. 1.148 per 1,000 d) and fewer revisions (0.159 *vs*. 0.581 per 1,000 d) The differences were not statistically significant (*p* = 0.22 and *p* = 0.18)	2	HeartMate II; Ventrassist	No patient-related predictors clearly analyzed; focus on mechanical trauma, micromotion, and driveline stabilization
Seretny *et al*. (2022) (Canada)^22^	Quality improvement project to reduce DLI and hospital admissions among LVAD patients within the first year after implantation	55 patients; median age 56 y. Non-CP group (n = 34); CP group (n = 21)	Quality Improvement (pre–post intervention study)	Incidence of first DLI within 1 y. Incidence of DLI during the initial hospitalization. Percentage of DLI-related hospital readmissions within the first year. Compliance score with the care pathway protocol	No patients in the CP group developed a DLI during the initial hospitalization compared to the non-CP group (18%). There was a trend toward fewer DLI-related readmissions in the CP group (55% *vs*. 67% in the non-CP group)	2	HVAD, HeartMate II, HeartMate 3	Obesity, age, diabetes, depression, renal failure, and device characteristics discussed as reported risk factors in prior literature; no independent patient-level predictor analysis
Tsuji *et al*. (2022) (Japan)^23^	To evaluate the safety and efficacy of prophylactic NPWT applied to the driveline exit site to prevent DLI within 1 y after LVAD implantation	100 patients; mean age 43.6 y. NPWT group (n = 50; mean age 42.3 years); SD group (n = 50; mean age 44.9 y)	Historically controlled cohort study	Primary: Incidence of DLI within 1 y of implantation Secondary: Safety, NPWT-related complications, all-cause mortality, and length of ICU and hospital stay	The incidence of DLI was 32% in the NPWT group and 42% in the standard dressing group (*p* = 0.30) NPWT was found to be safe, with no serious complications such as bleeding or infection directly associated with the device The anatomical shape of the DL exit site makes it difficult to maintain the airtight seal necessary for effective NPWT	2	DuraHeart, EVAHEART, Jarvik 2000, HeartMate II, HVAD, HeartMate 3	BMI, BSA, diabetes, albumin, creatinine, BNP, INTERMACS evaluated; no preoperative variables associated with DLI
Yoshitake *et al*. (2018) (Japan)^24^	To compare the effectiveness of the “triple tunnel method” *vs.* the traditional method for preventing DLI	34 patients; mean age 32.1 y. Group S (n = 14; mean age 31.7 y); group M (n = 20; mean age 32.3 y)	Retrospective cohort study	Incidence of DLI and 1 y freedom from readmission rate Group S (n = 14, subcutaneous route before 2014) Group M (n = 20, triple-tunnel method/muscle route since 2014)	Infection (DLI) occurred in 71.4% of group S compared with only 5% of group M (*p* < 0.05). Readmission for DLI at 1 y was 95% for group M compared with 64% for group S. Driveline location was significantly associated with the risk of infection (odds ratio 10.1)	2	HeartMate II; Jarvik 2000	Age, sex, and device type were evaluated; only driveline route remained significantly associated with DLI. BMI and diabetes were discussed as potential confounders but were not analyzed

The level of evidence was evaluated using the Oxford Centre for Evidence-Based Medicine (OCEBM) grading system.^27^ The classification criteria are as follows: 1 = Systematic review of randomized controlled trials (RCTs) or a single RCT; 2 = Systematic review of cohort studies, individual cohort studies, or low-quality RCTs; 3 = Systematic review of case–control studies or individual case–control studies; 4 = Case series or low-quality cohort and case-control studies; 5 = Expert opinion.

ACP, cold argon plasma; BMI, body mass index; BNP, brain natriuretic peptide; BSA, body surface area; CF-LVAD, continuous-flow left ventricular assist device; CKD, chronic kidney disease; COPD, chronic obstructive pulmonary disease; CP, care pathway; CPB, cardiopulmonary bypass; CRP, C-reactive protein; DL, driveline; DM, diabetes mellitus; eGFR, estimated glomerular filtration rate; EPPY, events per patient-year; FFP, fresh frozen plasma; HTN, hypertension; HM, HeartMate; HVAD, HeartWare Left Ventricular Assist Device; ICU, intensive care unit; LVEF, left ventricular ejection fraction; NYHA, New York Heart Association; NPWT, negative pressure wound therapy.

A visual summary of the preventive strategies and their reported clinical effects is presented in Figure 2. Sample size, duration of follow-up, and definitions of DLI varied substantially across studies, contributing to methodological heterogeneity. No randomized controlled trials were identified.

**Figure 2.**
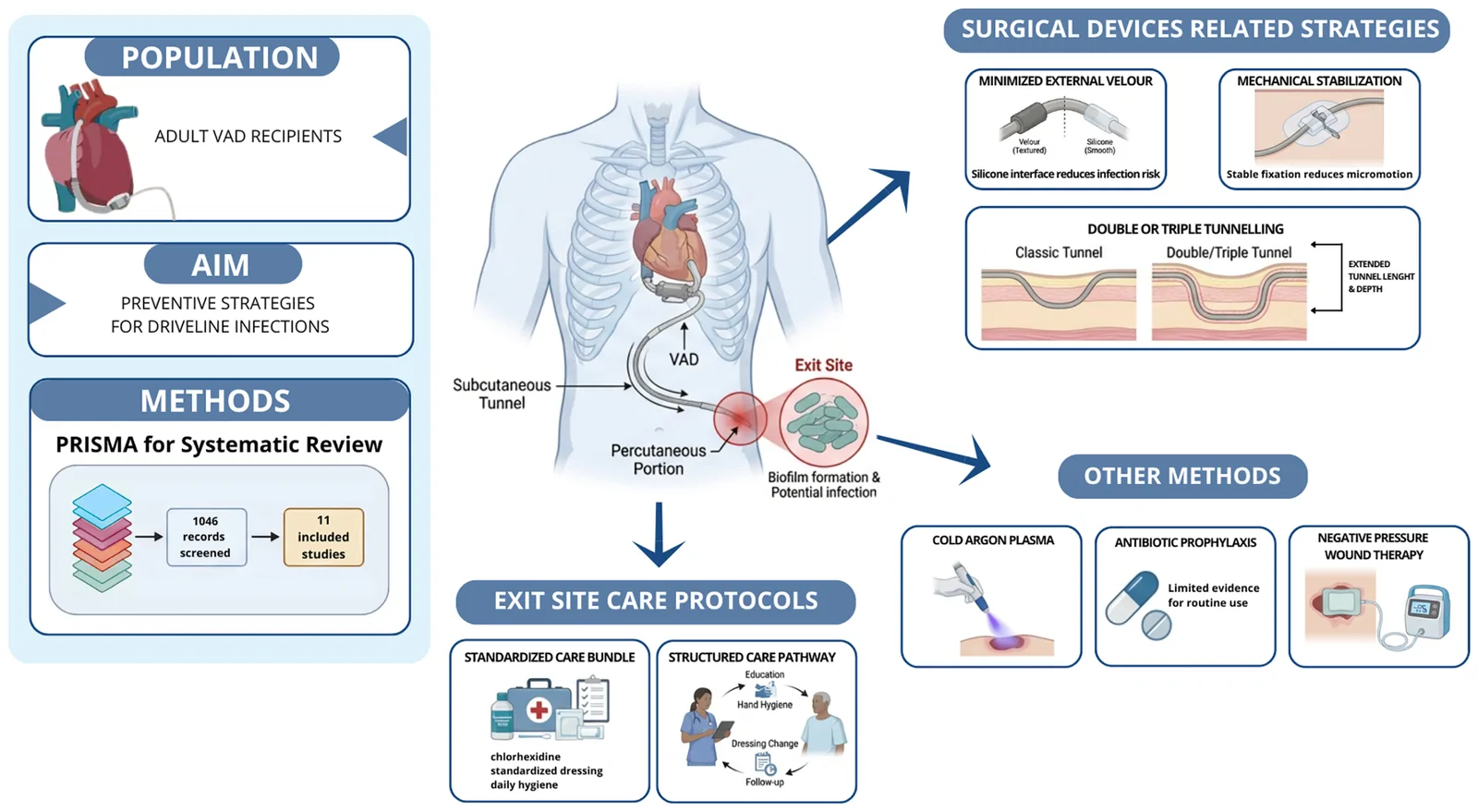
Visual summary of the preventive strategies and their reported clinical effects. VAD, ventricular assist device.

### Risk of Bias

Methodological quality was assessed using the JBI critical appraisal tools appropriate to each study design. Given that 10 of 11 included studies were observational,^14–24^ the cohort checklist was the most frequently applied. The results of the quality appraisal are summarized in Table 3.

**Table 3.** Critical Appraisal Checklists: Joanna Briggs Institute

Checklist for Cross-Sectional Studies														
Authors and Years	Q1	Q2	Q3	Q4	Q5	Q6	Q7	Q8				Results (%)		Level of Quality
Seretny *et al*. (2022)^22^	Y	Y	Y	Y	Y	U	Y	Y				87.5		High
**Checklist for Cohort Studies**														
**Authors and Years**	**Q1**	**Q2**	**Q3**	**Q4**	**Q5**	**Q6**	**Q7**	**Q8**	**Q9**	**Q10**	**Q11**	**Results (%**)		**Level of Quality**
Balestra *et al*. (2023)^14^	Y	Y	Y	Y	Y	Y	Y	Y	Y	N/A	Y	90	High	
Cagliostro *et al*. (2016)^15^	Y	Y	Y	U	Y	U	Y	Y	Y	Y	Y	81	High	
Camboni *et al*. (2017)^16^	U	Y	Y	Y	Y	Y	Y	Y	Y	Y	Y	90	High	
Durand *et al*. (2017)^17^	Y	Y	Y	U	Y	Y	Y	Y	Y	Y	Y	90	High	
Fleissner *et al*. (2013)^18^	Y	Y	Y	Y	Y	Y	Y	Y	Y	N/A	Y	90	High	
Lauenroth *et al*. (2023)^19^	Y	Y	Y	Y	Y	Y	Y	Y	Y	Y	Y	100	High	
Matsumoto *et al*. (2018)^20^	Y	Y	Y	Y	Y	Y	Y	Y	Y	N/A	Y	90	High	
Schibilsky *et al*. (2011)^21^	Y	Y	Y	Y	Y	Y	Y	Y	Y	N/A	Y	90	High	
Tsuji *et al*. (2022)^23^	Y	Y	Y	Y	Y	Y	Y	Y	Y	N/A	Y	90	High	
Yoshitake *et al*. (2018)^24^	Y	Y	Y	Y	Y	Y	Y	U	Y	N/A	Y	80	High	

Level of quality: Low <49%; moderate 50% to <69%; high ≥70%.

N, no; N/A, not applicable; U, unclear; Y, yes.

Overall, the methodological quality was judged to be high. Most studies clearly described patient populations, preventive interventions, and outcome assessment procedures.^14–16,18,24^ However, important limitations were consistently observed. The absence of randomization and allocation concealment across all studies introduced an inherent risk of selection bias.^17,19,21^ In several investigations, comparisons were based on historical controls or era-based analyses, increasing the risk of confounding due to evolving surgical techniques, device technology, and institutional learning curves.^15,18,24^ Adjustment for potential confounders was inconsistently performed. While some studies reported multivariable analyses or controlled comparisons,^20,23^ others relied primarily on unadjusted comparisons.^15,20^ Additionally, definitions and diagnostic criteria for DLI were not fully standardized across studies, contributing to heterogeneity in outcome reporting.^17,24^

Taken together, the body of evidence was characterized by moderate methodological rigor and corresponded to OCEBM Levels 2 and 3 of evidence. High-certainty evidence was not available due to the absence of randomized controlled trials and the inherent limitations of nonrandomized designs.

### Effects of Preventive Strategies on Driveline Infection

#### Surgical modifications of driveline routing and exit-site interface

Two studies evaluated surgical approaches aimed at reducing driveline micromotion and exit-site tissue trauma.^18,24^ A doubled driveline tunneling technique was associated with lower reported DLI compared with standard tunneling during follow-up.^18^ Similarly, a triple-tunnel method was associated with a markedly lower DLI occurrence compared with an alternative driveline route, with improved freedom from DLI-related readmission at 1 year.^24^ Overall, surgical routing modifications suggested a reduction in DLI risk compared with conventional approaches, although evidence was derived from observational designs and was frequently influenced by era-based comparisons.

#### Standardized care bundles and structured exit-site protocols

Implementation of standardized driveline care protocols was associated with improved infection outcomes. Cagliostro *et al*.^15^ reported that the introduction of a standardized driveline care kit reduced DLI event rates from 0.18 to 0.07 events per patient-year and significantly improved 1 year freedom from infection (log-rank *p* = 0.036).^15^ Seretny *et al*.,^22^ in a quality improvement study, found no DLI events during index hospitalization in patients managed under a structured care pathway compared with historical controls. Schibilsky *et al*.^21^ reported that structured exit-site management strategies were associated with improved infection control, although results were influenced by era-based comparisons. These findings suggest that protocolized standardization of processes and materials for exit-site management may reduce DLI burden.

#### Antiseptic and dressing management strategies

Balestra *et al*.^14^ evaluated antiseptic and dressing strategies in patients supported with HeartMate devices and reported variation in DLI rates depending on dressing materials and local care practices. Matsumoto *et al*.^20^ demonstrated that driveline exit angle and stabilization characteristics influenced DLI risk, with specific geometric configurations associated with lower infection incidence. Lauenroth *et al*.^19^ investigated the prophylactic treatment with argon cold plasma (ACP) applied for 30 days post-implant. The freedom from DLI was significantly higher in the ACP group at 30 days (100% *vs*. 85%; *p* = 0.012) and 6 months (92% *vs*. 69%; *p* = 0.007), although no differences were observed at 1 year (*p* = 0.65).^19^ These studies highlight the potential impact of local antiseptic measures and mechanical stability on short- and intermediate-term infection prevention.

#### Prophylactic NPWT

Tsuji *et al*.^23^ evaluated prophylactic NPWT at the driveline exit site. The 1-year DLI was 32% in the NPWT group *versus* 42% in the standard care group; however, this difference did not reach statistical significance (*p* = 0.30).^23^ Thus, while NPWT demonstrated feasibility, its preventive effectiveness was not clearly established in this cohort.

#### Antibiotic-based preventive strategies

Durand *et al*.^17^ evaluated prolonged topical polymyxin–trimethoprim prophylaxis in continuous-flow LVAD recipients. Chronic prophylaxis was associated with a significant reduction in DLI compared with standard care protocols.^17^ To date, no study has demonstrated a clear benefit of long-term systemic antibiotic prophylaxis as a standalone preventive measure.

#### Overall synthesis of clinical outcomes

Across all included studies, preventive strategies targeting mechanical stabilization, optimized surgical tunneling, and standardized exit-site care protocols were consistently associated with lower reported DLI incidence compared with conventional or historical practices. In contrast, adjunctive measures such as prophylactic NPWT or prolonged antibiotic prophylaxis demonstrated limited or inconsistent benefit. However, interpretation of these findings is constrained by the observational design of all included studies, heterogeneity in DLI definitions, variable follow-up duration, and the frequent use of historical or era-based comparators.

### Certainty of the Evidence

The certainty of evidence was assessed using the GRADE framework.^28^ As all included studies were observational, the body of evidence was initially rated as low certainty. Across intervention categories, further downgrading was applied due to risk of bias, reliance on historical or nonconcurrent comparators, heterogeneity in DLI definitions, and variability in follow-up duration. For surgical routing modifications and standardized care bundles, the certainty of evidence was judged as low to moderate, based on consistent direction of effect across independent cohorts. For adjunctive strategies, including prophylactic NPWT and antibiotic-based prophylaxis, the certainty of evidence remained low, given inconsistent findings and imprecision. No intervention achieved high-certainty evidence.

## Discussion

Driveline infection remains the most frequent LVAD-specific infectious complication and continues to represent a major cause of rehospitalization, prolonged antimicrobial therapy, and device-related morbidity and mortality.^29,30^ Despite advancements in continuous-flow technology, the presence of a percutaneous driveline creates a permanent disruption of the skin barrier, predisposing patients to colonization and biofilm formation.^30^ The presence of foreign material and a transcutaneous tract facilitate bacterial adherence and biofilm formation, most commonly involving gram-positive organisms such as *Staphylococcus aureus* and coagulase-negative staphylococci, although gram-negative pathogens are also reported.^29^

This systematic review demonstrates that preventive strategies targeting mechanical stabilization and surgical routing of the driveline are consistently associated with lower DLI incidence across observational cohorts. Modified tunneling techniques, reduced external velour exposure, and silicone-only exit-site interfaces were associated with significant reductions in infection rates in multiple independent studies.^16,18,24^ These findings align with the pathophysiological hypothesis that chronic micromotion and tissue shear stress contribute to persistent inflammation and bacterial entry at the driveline exit site.

The observed benefit of standardized care bundles is also consistent with broader infection prevention literature, where protocolization reduces variation in practice and improves outcomes.^9,11^ This is further supported by recent evidence emphasizing the importance of standardized driveline exit-site care and antiseptic protocols in reducing infection risk in LVAD populations.^4^ In LVAD populations specifically, exit-site management heterogeneity has been repeatedly identified as a contributor to DLI variability.^6^

Previous systematic reviews have emphasized the high incidence of DLI but have highlighted the lack of high-level evidence supporting specific preventive strategies.^6,8^ Our findings extend this knowledge by synthesizing more recent cohorts and identifying a consistent signal favoring mechanical and surgical optimization over pharmacological prophylaxis.

Importantly, the lack of consistent benefit from prolonged antibiotic prophylaxis observed in this review is congruent with prior concerns regarding antimicrobial resistance and biofilm persistence.^31^ Once biofilm is established along the driveline tract, systemic antibiotics alone are unlikely to eradicate infection, underscoring the importance of mechanical and structural preventive approaches. In selected cases of refractory or complex infection where device exchange is not feasible, heart transplantation may represent a salvage strategy.^3^ The evidence on antibiotic-based preventive strategies remains limited and heterogeneous. Perioperative prophylaxis regimens vary substantially across centers, most commonly including cephalosporins or vancomycin-based approaches, with broader-spectrum combinations used without clear evidence of superiority.^32^

Similarly, while NPWT has demonstrated benefit in other surgical wound contexts,^30,33^ its role in long-term DLI prevention remains uncertain. The absence of statistically significant reductions in DLI in comparative NPWT cohorts suggests that early wound optimization may not fully mitigate the chronic transcutaneous infection risk inherent to driveline support.^23^

The consistent association between reduced micromotion and lower infection rates supports a biomechanical model of DLI pathogenesis. Tissue traction and repeated exit-site manipulation may promote microfissures, local ischemia, and bacterial ingress. Stabilization devices, tunneling depth optimization, and minimization of velour exposure may therefore reduce both bacterial adhesion and inflammatory susceptibility.

Additionally, structured exit-site care bundles likely decrease cumulative handling and standardize antiseptic application. The benefit observed with chlorhexidine-based regimens in prior studies supports its continued use where tolerated,^33^ with evidence suggesting lower infection rates with chlorhexidine-based cleansing compared with povidone-iodine (5.4% *vs*. 42.9%).^11^

### Clinical Implications

Based on the synthesized evidence, preventive strategies for DLI should prioritize:

1. Surgical optimization of driveline routing
2. Minimization of external velour exposure
3. Mechanical stabilization of the driveline
4. Implementation of standardized, protocol-driven exit-site care

Routine long-term antibiotic prophylaxis cannot be supported by current evidence. Adjunctive technologies such as NPWT may be considered on a case-by-case basis but require further comparative evaluation.

### Strengths and Limitations

This review provides a focused synthesis of preventive strategies for DLI, incorporating recent international data and applying structured methodological appraisal (JBI, OCEBM, GRADE). However, the evidence base remains limited by observational design, heterogeneous definitions of DLI, inconsistent adjustment for confounders, and reliance on historical controls. The absence of randomized controlled trials significantly limits causal inference. In addition, differences in LVAD generations and driveline design across studies may have affected comparability, as device-specific characteristics could influence both mechanical stress and infection risk. Moreover, limited information was available regarding patient-related risk factors, including diabetes, obesity, frailty, and nutritional status, which may contribute to susceptibility to DLI and potentially influence the effectiveness of preventive strategies.

### Future Research Directions

Future research should prioritize developing multicenter prospective registries using standardized definitions of DLI. Comparative studies, including randomized or pragmatic controlled designs, are needed to evaluate different driveline routing techniques and preventive strategies. In addition, future investigations should incorporate objective assessments of driveline micromotion and tissue stress, as well as mechanistic studies focusing on biofilm formation and microbial colonization. Future studies should also evaluate the interaction between preventive interventions and patient-related risk factors to identify populations that may benefit from tailored approaches. Such efforts may help strengthen the evidence base and support the development of standardized recommendations for DLI prevention.

## Conclusions

Driveline infection remains a frequent and clinically significant complication in patients supported with VADs. Based on the available evidence, preventive strategies focused on surgical optimization of driveline routing, reduction of external velour exposure, mechanical stabilization, and implementation of standardized exit-site care protocols are consistently associated with lower reported DLI incidence. Conversely, adjunctive interventions such as prolonged antibiotic prophylaxis and prophylactic NPWT have not demonstrated consistent or durable benefit. However, the current evidence base is limited to observational studies and is characterized by methodological heterogeneity. No randomized controlled trials are available, and the overall certainty of evidence remains low to moderate, precluding definitive conclusions regarding the superiority of specific preventive strategies. Future research should prioritize standardized definitions, prospective multicenter designs, and controlled comparisons to establish definitive preventive standards for DLI.

## Supplementary Material


