# Supplementary material for: Preventive Strategies for Driveline Infections in Ventricular Assist Devices: A Systematic Review
Source: ASAIO J. 2026 Aug 3;72(9):721–32. doi: 10.1097/MAT.0000000000002800 (PMC13747280; doi:10.1097/MAT.0000000000002800)
Supplement: Supplementary file 1 [file mat-72-721-s001.pdf]

**Preventive Strategies for Driveline Infections in Ventricular Assist Devices: A Systematic Review**

**Supplementary files**

**Search strategy**

**PubMed (n= 280)**

| | |
|---|---|
| #1 | ("Heart-Assist Devices"[MeSH Terms] OR "ventricular assist device"[All Fields] OR "left ventricular assist device"[All Fields] OR "right ventricular assist device"[All Fields] OR "biventricular assist device"[All Fields] OR "LVAD"[All Fields] OR "RVAD"[All Fields] OR "BiVAD"[All Fields]) |
| #2 | ("Catheter-Related Infections"[MeSH Terms] OR "Surgical Wound Infection"[MeSH Terms] OR "Wound Infection"[MeSH Terms] OR "Infection"[All Fields] OR "driveline infection"[All Fields] OR "exit site infection"[All Fields] OR "device-related infection"[All Fields] OR "implant-associated infection"[All Fields] OR "wound infection"[All Fields]) |
| #3 | ("Infection Control"[MeSH Terms] OR "Primary Prevention"[MeSH Terms] OR "Antibiotic Prophylaxis"[MeSH Terms] OR "Chlorhexidine"[MeSH Terms] OR "preventive measures"[All Fields] OR "preventive strategy"[All Fields] OR "prevention"[All Fields] OR "infection prevention"[All Fields] OR "prophylaxis"[All Fields] OR "antibiotic prophylaxis"[All Fields] OR "chlorhexidine"[All Fields] OR "care protocol"[All Fields] OR "dressing change"[All Fields] OR "anchoring device"[All Fields] OR "nursing care"[All Fields] OR "nursing protocol"[All Fields]) |
| #4 | ("Heart-Assist Devices"[MeSH Terms] OR "ventricular assist device"[All Fields] OR "left ventricular assist device"[All Fields] OR "right ventricular assist device"[All Fields] OR "biventricular assist device"[All Fields] OR "LVAD"[All Fields] OR "RVAD"[All Fields] OR "BiVAD"[All Fields]) AND ("Catheter-Related Infections"[MeSH Terms] OR "Surgical Wound Infection"[MeSH Terms] OR "Wound Infection"[MeSH Terms] OR "Infection"[All Fields] OR "driveline infection"[All Fields] OR "exit site infection"[All Fields] OR "device-related infection"[All Fields] OR |

| | |
|---|---|
| | "implant-associated infection"[All Fields] OR "wound infection"[All Fields])<br>AND ("Infection Control"[MeSH Terms] OR "Primary Prevention"[MeSH Terms] OR<br>"Antibiotic Prophylaxis"[MeSH Terms] OR "Chlorhexidine"[MeSH Terms] OR<br>"preventive measures"[All Fields] OR "preventive strategy"[All Fields] OR<br>"prevention"[All Fields] OR "infection prevention"[All Fields] OR<br>"prophylaxis"[All Fields] OR "antibiotic prophylaxis"[All Fields] OR<br>"chlorhexidine"[All Fields] OR "care protocol"[All Fields] OR<br>"dressing change"[All Fields] OR "anchoring device"[All Fields] OR<br>"nursing care"[All Fields] OR "nursing protocol"[All Fields]) |

**CINAHL (n= 53)**

| | |
|---|---|
| #1 | (MH "Ventricular Assist Devices+") OR TI ("ventricular assist device*" OR<br>"left ventricular assist device" OR "right ventricular assist device" OR<br>"biventricular assist device" OR LVAD OR RVAD OR BiVAD) |
| #2 | MH "Catheter-Related Infections+") OR (MH "Surgical Wound Infections+") OR<br>(MH "Wound Infections+") OR (MH "Infections+") OR<br>TI ("driveline infection" OR "exit site infection" OR "device-related infection" OR<br>"implant-associated infection" OR "wound infection" OR infection) |
| #3 | (MH "Infection Control+") OR (MH "Primary Prevention+") OR<br>(MH "Antibiotic Prophylaxis") OR (MH "Chlorhexidine") OR<br>TI ("preventive measure*" OR "preventive strateg*" OR prevention OR<br>"infection prevention" OR prophylax* OR "antibiotic prophylaxis" OR<br>chlorhexidine OR "care protocol*" OR "dressing change*" OR<br>"anchoring device*" OR "nursing care" OR "nursing protocol*") |
| #4 | (MH "Ventricular Assist Devices+") OR TI ("ventricular assist device*" OR<br>"left ventricular assist device" OR "right ventricular assist device" OR<br>"biventricular assist device" OR LVAD OR RVAD OR BiVAD)<br>AND (MH "Catheter-Related Infections+") OR (MH "Surgical Wound Infections+") OR<br>(MH "Wound Infections+") OR (MH "Infections+") OR |

| | |
|---|---|
| | TI ("driveline infection" OR "exit site infection" OR "device-related infection" OR "implant-associated infection" OR "wound infection" OR infection) AND (MH "Infection Control+") OR (MH "Primary Prevention+") OR (MH "Antibiotic Prophylaxis") OR (MH "Chlorhexidine") OR TI ("preventive measure*" OR "preventive strateg*" OR prevention OR "infection prevention" OR prophylax* OR "antibiotic prophylaxis" OR chlorhexidine OR "care protocol*" OR "dressing change*" OR "anchoring device*" OR "nursing care" OR "nursing protocol*") |

**Web of Science (n=219)**

| | |
|---|---|
| #1 | ("ventricular assist device*" OR "left ventricular assist device" OR "right ventricular assist device" OR "biventricular assist device" OR LVAD OR RVAD OR BiVAD) |
| #2 | ("driveline infection" OR "exit site infection" OR "device-related infection" OR "implant-associated infection" OR "wound infection" OR infection) |
| #3 | ("infection control" OR "primary prevention" OR "antibiotic prophylaxis" OR chlorhexidine OR "preventive measure*" OR "preventive strateg*" OR prevention OR "infection prevention" OR prophylax* OR "care protocol*" OR "dressing change*" OR "anchoring device*" OR "nursing care" OR "nursing protocol*") |
| #4 | ("ventricular assist device*" OR "left ventricular assist device" OR "right ventricular assist device" OR "biventricular assist device" OR LVAD OR RVAD OR BiVAD) AND ("driveline infection" OR "exit site infection" OR "device-related infection" OR "implant-associated infection" OR "wound infection" OR infection) AND ("infection control" OR "primary prevention" OR |

| | |
|---|---|
| | "antibiotic prophylaxis" OR chlorhexidine OR<br>"preventive measure*" OR "preventive strateg*" OR prevention OR<br>"infection prevention" OR prophylax* OR "care protocol*" OR<br>"dressing change*" OR "anchoring device*" OR<br>"nursing care" OR "nursing protocol*") |

**Scopus (n= 396)**

| | |
|---|---|
| #1 | (TITLE-ABS-KEY("ventricular assist device*" OR<br>"left ventricular assist device" OR "right ventricular assist device" OR<br>"biventricular assist device" OR LVAD OR RVAD OR BiVAD) |
| #2 | TITLE-ABS-KEY("driveline infection" OR "exit site infection" OR<br>"device-related infection" OR "implant-associated infection" OR<br>"wound infection" OR infection) |
| #3 | TITLE-ABS-KEY("infection control" OR "primary prevention" OR<br>"antibiotic prophylaxis" OR chlorhexidine OR<br>"preventive measure*" OR "preventive strateg*" OR prevention OR<br>"infection prevention" OR prophylax* OR "care protocol*" OR<br>"dressing change*" OR "anchoring device*" OR<br>"nursing care" OR "nursing protocol*")) |
| #4 | (TITLE-ABS-KEY("ventricular assist device*" OR<br>"left ventricular assist device" OR "right ventricular assist device" OR<br>"biventricular assist device" OR LVAD OR RVAD OR BiVAD)<br>AND TITLE-ABS-KEY("driveline infection" OR "exit site infection" OR<br>"device-related infection" OR "implant-associated infection" OR<br>"wound infection" OR infection)<br>AND TITLE-ABS-KEY("infection control" OR "primary prevention" OR<br>"antibiotic prophylaxis" OR chlorhexidine OR<br>"preventive measure*" OR "preventive strateg*" OR prevention OR<br>"infection prevention" OR prophylax* OR "care protocol*" OR |

| | |
|---|---|
| | "dressing change*" OR "anchoring device*" OR<br><br>"nursing care" OR "nursing protocol*")) |

**Cochrane Library (n= 94)**

| | |
|---|---|
| #1 | ("Heart-Assist Devices" OR "ventricular assist device*" OR<br><br>"left ventricular assist device" OR "right ventricular assist device" OR<br><br>"biventricular assist device" OR LVAD OR RVAD OR BiVAD) |
| #2 | ("Catheter-Related Infections" OR "Surgical Wound Infection" OR<br><br>"Wound Infection" OR "driveline infection" OR "exit site infection" OR<br><br>"device-related infection" OR "implant-associated infection" OR<br><br>"wound infection" OR infection) |
| #3 | ("Infection Control" OR "Primary Prevention" OR<br><br>"Antibiotic Prophylaxis" OR "Chlorhexidine" OR<br><br>"preventive measure*" OR "preventive strateg*" OR prevention OR<br><br>"infection prevention" OR prophylax* OR<br><br>"care protocol*" OR "dressing change*" OR<br><br>"anchoring device*" OR "nursing care" OR "nursing protocol*") |
| #4 | ("Heart-Assist Devices" OR "ventricular assist device*" OR<br><br>"left ventricular assist device" OR "right ventricular assist device" OR<br><br>"biventricular assist device" OR LVAD OR RVAD OR BiVAD)<br><br>AND ("Catheter-Related Infections" OR "Surgical Wound Infection" OR<br><br>"Wound Infection" OR "driveline infection" OR "exit site infection" OR<br><br>"device-related infection" OR "implant-associated infection" OR<br><br>"wound infection" OR infection)<br><br>AND ("Infection Control" OR "Primary Prevention" OR<br><br>"Antibiotic Prophylaxis" OR "Chlorhexidine" OR<br><br>"preventive measure*" OR "preventive strateg*" OR prevention OR<br><br>"infection prevention" OR prophylax* OR<br><br>"care protocol*" OR "dressing change*" OR |

| | |
|---|---|
| | "anchoring device*" OR "nursing care" OR "nursing protocol*") |

**APA PsycInfo (n = 4)**

| | |
|---|---|
| #1 | TX ("ventricular assist device" OR<br>"left ventricular assist device" OR "right ventricular assist device" OR<br>"biventricular assist device" OR LVAD OR RVAD OR BiVAD) |
| #2 | TX ("driveline infection" OR "exit site infection" OR<br>"device-related infection" OR "implant-associated infection" OR<br>"wound infection" OR infection) |
| #3 | TX ("infection control" OR "primary prevention" OR<br>"antibiotic prophylaxis" OR chlorhexidine OR<br>"preventive measure*" OR "preventive strateg*" OR prevention OR<br>"infection prevention" OR prophylax* OR<br>"care protocol*" OR "dressing change*" OR<br>"anchoring device*" OR "nursing care" OR "nursing protocol*") |
| #4 | TX ("ventricular assist device" OR<br>"left ventricular assist device" OR "right ventricular assist device" OR<br>"biventricular assist device" OR LVAD OR RVAD OR BiVAD)<br>AND TX ("driveline infection" OR "exit site infection" OR<br>"device-related infection" OR "implant-associated infection" OR<br>"wound infection" OR infection)<br>AND TX ("infection control" OR "primary prevention" OR<br>"antibiotic prophylaxis" OR chlorhexidine OR<br>"preventive measure*" OR "preventive strateg*" OR prevention OR<br>"infection prevention" OR prophylax* OR<br>"care protocol*" OR "dressing change*" OR<br>"anchoring device*" OR "nursing care" OR "nursing protocol*") |

| | |
|---|---|
| Total records retrieved | 1041 |
